# An Analytical Investigation of Anomaly Detection Methods Based on Sequence to Sequence Model in Satellite Power Subsystem

**DOI:** 10.3390/s22051819

**Published:** 2022-02-25

**Authors:** Weihua Jin, Shijie Zhang, Bo Sun, Pengli Jin, Zhidong Li

**Affiliations:** 1 Research Center of Satellite Technology, Harbin Institute of Technology, Harbin 150080, China; wh.jin@hotmail.com; 2 Beijing Institute of Spacecraft System Engineering, Beijing 100094, China; lizhidongcas@163.com; 3 School of Materials Science & Engineering, Harbin Institute of Technology, Harbin 150080, China; pljin@foxmail.com

**Keywords:** satellite power subsystem, anomaly detection, sequence to sequence

## Abstract

The satellite power subsystem is responsible for all power supply in a satellite, and is an important component of it. The system’s performance has a direct impact on the operations of other systems as well as the satellite’s lifespan. Sequence to sequence (seq2seq) learning has recently advanced, gaining even more power in evaluating complicated and large-scale data. The potential of the seq2seq model in detecting anomalies in the satellite power subsystem is investigated in this work. A seq2seq-based scheme is given, with a thorough comparison of different neural-network cell types and levels of data smoothness. Three specific approaches were created to evaluate the seq2seq model performance, taking into account the unsupervised learning mechanism. The findings reveal that a CNN-based seq2seq with attention model under suitable data-smoothing conditions has a better ability to detect anomalies in the satellite power subsystem.

## 1. Introduction

The satellite power subsystem converts solar energy into electrical energy to provide power for the satellite’s routine operation. The normal operation of the power subsystem is very important to the satellite [1]. As the core of the satellite, it directly affects whether the whole satellite can work. Due to the special working environment, the proportion of satellite power subsystem failures has reached 30%, according to statistics of 300 cases of on-orbit spacecraft failures from 1993 to 2012 [2]. There are many sensors in the power subsystem to monitor its working status. These sensors generate a vast quantity of time-series data, known as the telemetry data, which are relayed back to the ground, where experts may evaluate it to see if the power subsystem is malfunctioning [3]. However, there are relatively few anomalous samples of satellites in orbit, which is not enough to support supervised training. Therefore, anomaly detection in the power subsystem has become a problem of how to find anomalies from multivariate time series without supervision [4,5]. The common method is to model normal patterns and detect anomalies by identifying patterns that rarely occur [6]. The power subsystem is a complex nonlinear system, and it is difficult to establish an accurate mathematical model. The development of machine learning provides a good method, as it has a strong nonlinear fitting ability when the amount of data is sufficient, so it will play a very important role in the anomaly detection in satellite power subsystems.

The unsupervised anomaly detection of multivariate time series has been widely studied in existing works. The two most common types of anomalies are point anomalies and pattern anomalies. A point anomaly looks for an uncommon data point (time point) in a time series, whereas a pattern anomaly finds an unexpected pattern of changes (time sequence) [5]. Many traditional algorithms are used for unsupervised point anomaly detection. These key approaches include One-Class Support Vector Machines (OCSVM), Principal Component Analysis (PCA), and K-means algorithm (KM) [1,5,7,8]. As with traditional SVM, OCSVM is a binary classification method, but the training samples of OCSVM are only positive. Schölkopf B et al. first introduced the idea of OCSVM [9]. When the boundary of normal data is obtained through training, the target outside the boundary can be determined as an anomaly [10,11,12]. Erfani S M et al. presented a hybrid model where an OCSVM is trained from the features learned by the deep belief networks (DBN), which are trained to extract generic underlying features. This hybrid model is used to solve high-dimensional and large-scale anomaly detection problems [10]. Although it can achieve good results, the training of the DBN network and SVM is time-consuming. It is not applicable for the large-scale and multivariate time-series satellite power subsystem data [13,14,15]. The application scenario of PCA is to reduce the dimension of the data set, and the reduced dimension data can retain the characteristics of the original data to the greatest extent. The eigenvector obtained by PCA after eigenvalue decomposition reflects the different directions of the variance change degree of the original data, and the eigenvalue is the variance of the data in the corresponding direction. If the eigenvalue of a single data sample is not consistent with the overall data sample, for example, if it deviates greatly from other data samples in some directions, it may indicate that the data sample is an anomalous point [16,17,18]. Bo Lee and Xingsheng Wang established the approximate PCA model for the satellite power system, by which the target can be determined as an anomaly by detecting whether squared prediction error (SPE) and sensor validity index (SVI) exceed the confidence limit [16]. However, the PCA principle is mainly used to eliminate the correlation between variables, and assuming that this correlation is linear, it is difficult to obtain good results for non-linear dependencies with this model. K-means (KM) algorithm is the most commonly used clustering-based algorithm. Its principle is to divide the data into a predetermined number of classes K based on minimizing the error function. When a target does not belong to any class, it will be judged as an anomaly [19,20,21]. The KM algorithm is relatively simple in principle, easy to implement and fast in convergence. However, when the number of features increases, the effect of clustering also decreases.

For the detection of pattern anomalies, the simplest way is by counting the number of mismatches. Mismatches are subsequences in a test sequence that do not appear in the standard data. An anomaly is defined as a test sequence with a substantial number of mismatches [5,22,23,24]. Rather than looking for exact matches, soft mismatch scores can also be computed. Terran L and Carla E presented an approach to transform the temporal sequences of discrete, unordered observations into a metric space via similarity measure (1-nearest-neighbor classification rule) that encodes intra-attribute dependencies. Classification boundaries are selected from a posteriori characterization, coupled with the greedy clustering technique. This method has good results when the number of features is small, but when the number of features increases, it will suffer the curse of dimensionality, and the method requires many parameters to be set manually [25,26]. An alternative detection strategy is as follows: the time series *T* is broken into multiple subsequences, and the subsequence *D* is said to be the anomaly of *T* if *D* has the largest distance to its nearest non-overlapping match [5,27]. This approach requires the calculation of the distance between all possible subsequences and the sequence to be detected. Top-K pruning can be used to make this computation efficient. Although there are some other methods to reduce the amount of computation, this anomaly detection method is also mechanical and computationally time-consuming.

To summarize, these above traditional machine learning methods are highly dependent on the quality of data and aren’t suitable for anomaly detection in satellite power subsystems. The main challenges are summarized as follows: (1) The number of features related to anomaly detection of power subsystems exceeds more than 30, and the use of traditional machine learning methods will suffer the curse of dimensionality. In addition, since there are many types of anomaly in the power subsystem and the causes of the anomaly are complex, if the dimensionality reduction algorithm is used, it will inevitably cause the loss of some anomalous information. (2) When moving around the earth, the satellite keeps entering and leaving the earth’s shadow, which leads to the continuous transition of the working conditions of the satellite power subsystem. The changes in power consumption of satellite loads will also lead to the switching of working conditions. When the working conditions change, the telemetry value usually changes suddenly, which could lead to a false alarm. False alarms greatly increase the workload of ground operations managers. (3) Satellite power subsystem telemetry data contain time information, and traditional methods cannot make good use of this information. They do not take into account the long-term and short-term dependency problem present in time series data, or deal with time-series data in accordance with ordinary-series data. For time-dependent anomalies, traditional methods cannot effectively detect them.

One of the solutions for the first challenge is the autoencoder. It is a type of recurrent neural network that is trained to reconstruct the input sequence. The autoencoder is first trained with normal data in order to detect anomalies. When unseen pattern data are fed into the model, the model is unable to rebuild unseen pattern data with the same level of quality as normal pattern data reconstruction [1,28,29,30,31,32]. Weihua Jin et al. proposed a novel stage-training denoising autoencoder (ST-DAE) which trains the feature in stages to reduce the false alarm rate [1]. Pankaj Malhotra et al. proposed a Long Short-Term Memory (LSTM) networks-based autoencoder scheme for anomaly detection that learns to reconstruct normal time-series data and then uses reconstruction error to detect anomalies [31].

However, for the latter two challenges, traditional autoencoders cannot cope very well. With the progress of technology, the autoencoder has also undergone significant development. An important autoencoder variant structure is seq2seq, whose input and output data are sequences. Seq2seq learning is widely used in statistical machine translation, and has begun to show promising results. Seq2seq learning has a strong ability to extract high-level features, and the implementation of the seq2seq model can take into account the time information of the features. In the field of medical diagnosis, seq2seq model has been used for patient arrhythmia detection from ECG heartbeat data [33,34]. There is a lack of studies to systematically examine the potential of seq2seq learning in the unsupervised anomaly detection of satellite power subsystems. The seq2seq-based ensemble scheme has been developed considering different neural network cell types and the level of data smoothness. Furthermore, novel methods have been developed to evaluate the seq2seq model’s performance. The paper is organized as follows: Section 2 introduces the materials. Section 3 presents the research methodology. The experiments and results will be shown and discussed in Section 4. Section 5 presents the conclusions.

## 2. Materials

### 2.1. Satellite Power Subsystem

**Figure 1:**
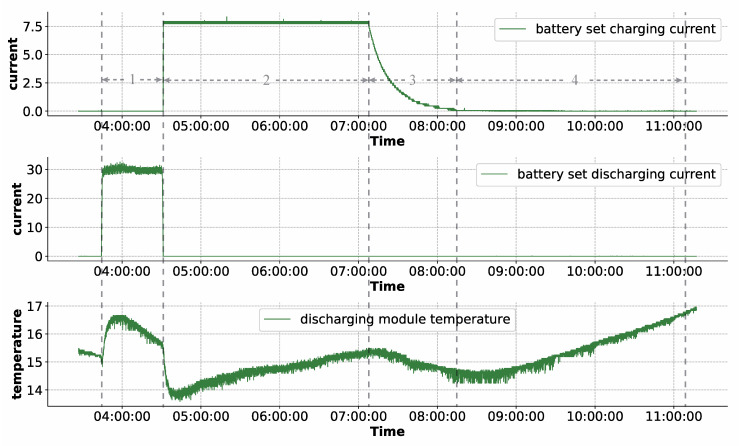
Four kinds of working conditions division of satellite power subsystem.

The satellite power subsystem consists of three main components, a pair of solar panels, two battery sets, and the Power Control Unit (PCU). The satellite alternately enters light and shadow zones as it travels around the Earth. According to the light conditions of satellite operation and the changes of the battery set charging and discharging, the working conditions of the satellite power subsystem can be roughly divided into four types, namely, Discharging Domain, Full Charging Domain, Trickle Charging Domain, and Shunting Domain. Figure 1 shows the four working conditions divided by the battery set charging current, the battery set discharging current, and the shunt module temperature. The Discharging Domain is represented by ‘1’ in the Figure 1. In this working state, the satellite is in the Earth’s shadow zone, fully unable to be illuminated. As a result, solar panels are unable to generate electricity, and the spacecraft requires battery set discharge to assure normal operation. The charging current of the battery is in the smallest range, while the discharge current is in the maximum range, as illustrated in Figure 1; The Full Charging Domain is represented by ‘2’ in the figure. The satellite swiftly enters the light zone from the Earth’s shadow zone under this working condition, the solar panels are exposed to adequate light, and the solar panels generate enough electrical energy, which is partly delivered to the spacecraft and partly utilized to charge the battery at full power. The battery set’s charging current is at its maximum, while the discharge current is at its lowest. The Trickle Charging Domain is represented by the ‘3’ in the figure. When the battery set is nearly fully charged under this working condition, the charging power must be lowered. The charging current of the battery drops from its maximum value to zero, as illustrated in Figure 1, and the discharge current is in the minimal range; The zone represented by ‘4’ in the figure is the Shunting Domain. Under this working condition, the power generated by the solar array is sufficient to power the satellite, and the battery pack is also fully charged, so the charging and discharging currents of the battery pack are zero, however, the excess power generated by the solar array needs to be released through the shunt module, so the temperature of the shunt module rises. The satellite power subsystem works alternately with these four working conditions. It can be seen that the working conditions change relatively quickly, which is the main reason for false alarms.

### 2.2. Sequence to Sequence Model

Seq2seq is a network of encoder-decoder structures. Its input and output are both sequences. The encoder transforms a variable-length signal sequence into a fixed-length vector expression, and the decoder transforms the fixed-length vector into a variable-length target signal sequence [32,35]. There are many ways to implement the seq2seq model.

One implementation is the Seq2seq model based on Recurrent Neural Networks (RNN). Both encoder and decoder adopt RNN series models, generally LSTM, GRU [35], etc. Ilya Sutskever et al. proposed a multi-layered LSTM to map the input sequence to a vector of fixed dimensionality, and then another deep LSTM to decode the target sequence from the vector to deal with these problems, whose input and output sequences have different lengths with complicated relationships. In addition, they found it extremely valuable to reverse the order of the data in the input sentence [32]. Kyunghyun Cho et al. proposed a Gated Recurrent Unit (GRU), which is a variant of LSTM [35,36]. The seq2seq framework based on RNN first encodes the input sequence into a fixed-size vector. This process is a process of information compression, which inevitably loses a lot of information. Moreover, the decoder cannot pay attention to more details of the input sequence during decoding, which leads to the attention mechanism, which solves the problem by imitating human attention. Bahdanau D et al. used attention mechanism for the first time in the field of Natural Language Processing (NLP), and proposed that the bottleneck of machine translation using encoder–decoder is that no matter how long the input is, the common practice is to combine all the inputs into a fixed-length vector to represent the sentence by some method, which causes the problem that if the sentence is very long, such a method will not work very well as the input to the decoder [37]. The attention model differs from the encoder–decoder model in that it does not need the encoder to encode all of the input data into a single fixed-length vector. Instead, the encoder converts the input into a sequence of vectors, with a subset of the vector sequence being picked for further processing at each step of the decoding process. As a result, each output is created in such a way that it fully utilizes the data conveyed by the input sequence. In translation assignments, this strategy has produced excellent results [38,39]. After the attentional mechanism was proposed, a number of variants emerged. Luong M T et al. proposed two variants of the attention mechanism, namely the global attentional model and the local attentional model [40]. Vaswani A et al. proposed the self-attention mechanism and a new simple network architecture, the Transformer, based solely on attention mechanisms [39].

Another important implementation is the seq2seq model based on Convolutional Neural Networks (CNN). In the convolutional seq2seq model, the convolution sequence length of each layer is unchanged through padding. This operation can ensure that the sequence length is consistent in the multi-layer convolution network [41]. The convolution layer is successfully applied to seq2seq tasks, which gives play to the advantages of CNN’s parallel computing and hierarchical structure. The parallel computing of the convolution layer improves the running speed. At the same time, the hierarchical structure of CNN makes it convenient for the model to find the structural information in sentences [42,43,44]. It is also possible to use multi-step attention to connect encoder and decoder, i.e., to calculate attention separately for each layer of the decoder.

## 3. Research Methodology

**Figure 2:**
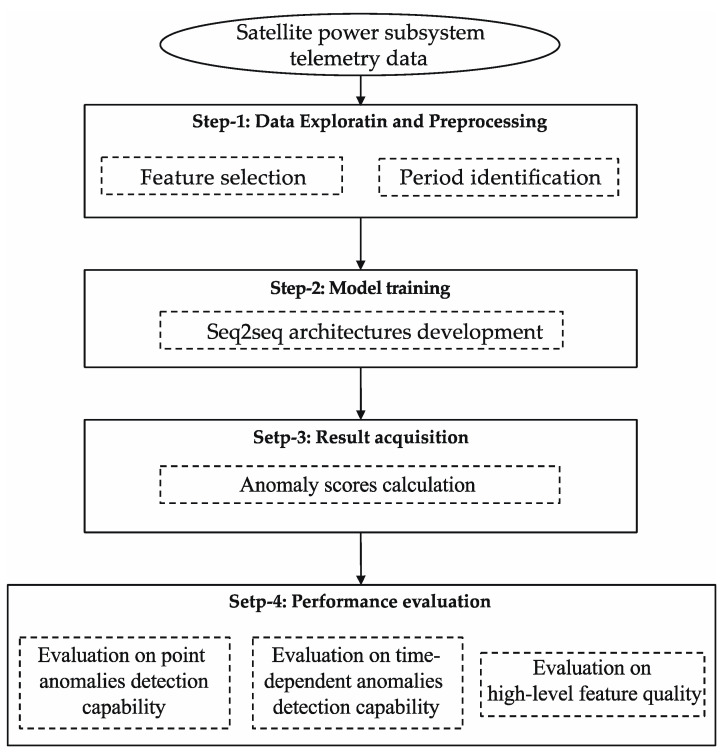
Research outline.

The proposed method is shown in Figure 2. This approach consists of four steps to conduct the analytical investigation: data exploration and preprocessing, model training, result acquisition, and performance evaluation.

### 3.1. Data Exploration and Preprocessing

Data exploration is the initial and important process for understanding the characteristics of telemetry data from the satellite power subsystem. The first step is feature selection, which is very important for determining the most influential variables indicating the health status of the satellite. Because each variable has practical significance, the best way to filter it is with expert knowledge.

The second step is to identify dominant periods in the telemetry data of the satellite power subsystem. The length of the single dominant period can help us determine the length of the sequence input to the seq2seq model. As described in Section 2.1, these four working conditions vary in cycles as the satellite travels around the Earth, and the length of their respective working time varies. For the model to extract the complete features, the length of the sequence must be greater than the maximum length of the working condition.

The majority of satellite power subsystem telemetry data are voltage and current, which are volatile and may be considered as noise by the neural network when trained, preventing the neural network from converging. We could deal with these features using the moving-average method to fix this problem [1,45], which can be calculated using:

(1) $$E_{t}=\frac{A_{t-1}+A_{t-2}+A_{t-3}+\cdots +A_{t-n}\;}{n},$$

where $A_{t-n}$ represents actual values for the previous period. n is window size of moving average method. The value of n largely affects smoothing and denoising, and this paper will test the effects of different values of n on the effect of seq2seq model.

### 3.2. Development of Seq2seq-Based Scheme

As summarized in Table 1, 12 individual seq2seq frameworks were developed considering different neural network cell types and the window size of moving average method. LSTM, LSTM with attention, CNN, and CNN with attention were considered, and the resulting seq2seqs are denoted with ‘LSTM’, ’LSTM-a’, ’CNN’, and ‘CNN-a’ respectively. The attention mechanism used by the LSTM network is similar to that in the paper of Ref. [28], and that used by the CNN network is similar to that in the paper of Ref. [32]. ‘N’ represents the window size of moving average method, which is determined in order to investigate the influence on anomaly detection capacity of seq2seq frameworks. Five-length levels are considered, i.e., 1, 2, 4, 6, and 8.

### 3.3. Result Acquisition

After the training of the seq2seq model, we needed to obtain the corresponding results for later evaluation. The seq2seq model is also a kind of autoencoder. We can calculate the reconstruction error as the basis for evaluation. The most common calculation method for reconstruction error is the root mean square error, which can be calculated using:

(2) $$e=\sqrt{\frac{\sum_{i=1}^{n}{\left(x_{i}-x_{i}^{\prime }\right)}^{2}}{s}},$$

where s is the sequence length [6]. Reconstruction errors need to be normalized, as the reconstruction results are obtained using different models. We can obtain normalized errors by Equation (3):

(3) $$e^{\prime }=\frac{e_{i}-e_{min}}{e_{max}-e_{min}},$$

Reconstruction errors can be regarded as anomaly scores, which are used to indicate the degree of abnormality of the samples.

### 3.4. Performance Evaluation

Due to a lack of anomaly samples, evaluating the performance of the described above seq2seq models is challenging. The proposed seq2seq models are assessed from three angles in this study and the ST-DAE [1] is used as the baseline method. First and foremost, the seq2seq model is essentially an encoder–decoder network, which must have strong input-data-reconstruction ability, and the false alarm rate can be used to evaluate this ability. Models with stronger reconstruction skills are likely to have a lower false alarm rate. In other words, when models don’t extract all of the features of the normal data, a false alarm rate arises. To calculate the false alarm rate, we first need to determine the anomaly threshold. The anomaly scores can be grouped into two clusters using the K-means clustering algorithm, with the anomaly threshold being the minimum anomaly score of the anomaly cluster [1]. In addition, in satellite power subsystems, random point anomalies, i.e., outliers, were common. The main causes of random point anomalies are data transmission between satellites and earth, the complex electromagnetic environment in space and the switching of main and standby equipment in satellites. This anomaly is more common than other serious anomalies. The reconstruction ability of the model can also be evaluated by the detection rate of point anomalies.

**Figure 3:**
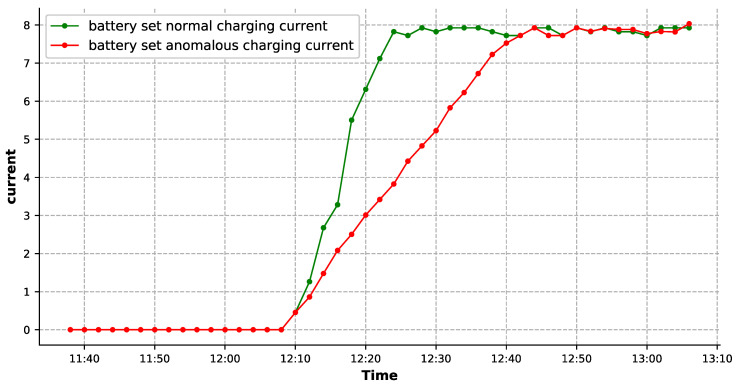
Battery set normal and anomalous charging current.

Secondly, a time-dependent anomaly is not an anomaly from the perspective of a single moment in time, but rather from the perspective of a period of time. When the satellite power subsystem operates correctly, the solar panel can swiftly generate electricity to charge the battery set, as illustrated in Figure 3, and the battery set charging current will rapidly grow. When the satellite power subsystem’s operation is anomalous, the rate of rise of the battery charging current is lowered. The traditional autoencoders, such as denoising autoencoder and Sparse Autoencoder, cannot effectively detect this kind of time-dependent anomaly. The seq2seq model’s performance can be evaluated based on the detection capability of this time-dependent anomaly.

Thirdly, the seq2seq models’ performance can be evaluated based on the quality of high-level features extracted. As described in Section 2.1, the working conditions of the satellite power subsystem can be roughly divided into four types. High-level features should be able to classify the data into these four types. We performed unsupervised clustering on the extracted high-level features after the model was trained, and the quality of the high-level features was determined by evaluating the clustering effect. The Silhouette Coefficient [46] and the Calinski-Harabasz Index [47] are two often-used metrics for assessing the clustering effect. The equations are briefly defined here as:

The Silhouette Coefficient score is given by Equation (4):

(4) $$s=\frac{b-a}{\max\left(a,b\right)},$$

where *a* is the mean distance between a sample and all other points in the same class and *b* is the mean distance between a sample and all other points in the next nearest cluster [48]. The Silhouette Coefficient score ranges from −1 to +1 for faulty clustering to highly dense clustering. Overlapping clusters are indicated by scores near 0.

The Calinski-Harabasz Index is given by Equation (5):

(5) $$CH=\left[\frac{\sum_{k=1}^{K}n_{k}{\left\|c_{k}-c\right\|}^{2}}{K-1}]/[\frac{\sum_{k=1}^{K}\sum_{i=1}^{n_{k}}{\left\|d_{i}-c_{k}\right\|}^{2}}{N-K}\right],$$

where $n_{k}$ and $c_{k}$ denote the number of points and centroid of the kth cluster, c is the global centroid, and N is the total number of data points. When clusters are dense and well separated, the score is greater, which corresponds to a standard cluster concept.

## 4. Experiments and Discussions

### 4.1. Description of The Satellite Power Subsystem Telemetry Data

The methodology is applied to analyze the real telemetry data of a navigation satellite power subsystem. There are 125486 rows and 65 variables in the original data set. These variables can be generally classified into three types: (1) voltage features (i.e., bus voltage, whole battery set voltage, single battery cell voltage, and main error amplifier voltage); (2) current features (i.e., bus current, solar cell array current, battery set charging current, battery set discharging current, battery charging regulator input current and battery discharging regulator output current); (3) temperature features (i.e., charging module temperature and discharging module temperature).

### 4.2. Data Preprocessing

In the data preprocessing stage, the first step is feature selection. In the paper, we selected meaningful features based on expert knowledge. According to expert knowledge, the following four types of features can be removed: (1) backup features: the backup values of the important features, such as the bus current and battery set charge current; (2) low-frequency sampled features: the values are used to verify high-frequency sampled features; (3) switch features: the values are almost unchanged and ground command is required to change, such as battery charge regulator switch; (4) flag features: for example, battery charging overvoltage protection flag, which marks when there is a serious fault in the satellite [1]. After feature selection and removing rows with excessive missing values, a dataset with 36 features and 124,116 rows was generated. The 36 features included bus current, battery set charge current, battery set discharge current, battery charge regulator input current, battery charge regulator output current, battery temperature, solar cell array current, battery set whole voltage, battery set single voltage, main error amplifier voltage and battery error amplifier voltage.

Another important task was to determine the length of the sequence. As described in Section 2.1, the satellite power subsystem always switches between four different working conditions. The division of working conditions helps to determine the sequence length. The length of the sequence must be longer than the maximum period of the working condition for the model to extract all of the features. According to data, this length is estimated to be about 240. Furthermore, we can see that we cannot distinguish between the four working conditions based on one feature alone. We expect the high-level features extracted by the seq2seq model to accurately distinguish between these four working conditions.

The third step of data preprocessing was to process the data according to Equation (1) and finally obtain five data sets. When the length of the moving average window is 4, 6 and 8, the data need to be processed into multiple subsequences with overlap [5]. The size of the overlap is half the length of the moving average window. Ultimately, these data can be used as direct input to the model.

### 4.3. Model Training

As summarized in Table 2, 12 individual seq2seq frameworks were developed. The detailed configurations of seq2seq models are shown in Table 3 and Table 4. To facilitate comparison of the effects of different neural network cell types on the results, the experiments used roughly the same network structure, both extracting two high-level features.

In the experiment, the common activation functions were tested. After the experiments, the LSTM-based model worked better with the Tanh activation function, while the CNN-based model worked better with the Relu activation function. Bayesian optimization was used to find the best values for the other hyperparameters, such as batch size and learning rate. The search ranges were [4, 8, 16, 32, 64, 128, 256] and [$10^{-4}$, $10^{-3},\;10^{-2},\;10^{-1}$], respectively. After the model training was completed, the anomaly score was calculated based on Equations (2) and (3) according to Section 3.3.

### 4.4. Performance Evaluation

#### 4.4.1. Evaluation on Model’s Reconstruction Capability

**Figure 4:**
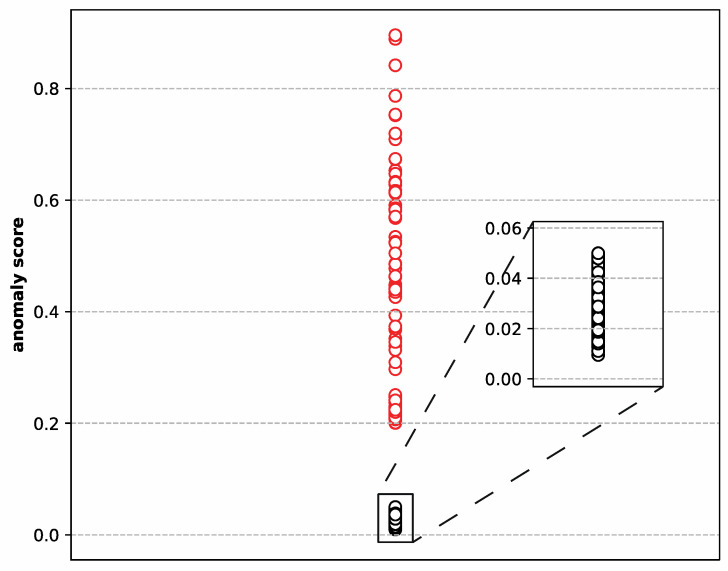
The clustering result of the anomaly scores of the ‘LSTM-1’ model.

A well-developed seq2seq model must have a strong ability to capture the intrinsic data behavior and reconstruct the input sequence data. The transitions between various working conditions for satellite power subsystems are quick, which can cause abrupt changes in numerous features in the telemetry data, as shown in Figure 1. It is challenging to model these behaviors at the junction of these changing working conditions, and they are prone to false alarms. Therefore, the number of false alarms caused by the model can be used to assess the model’s capacity to reconstruct. According to the method mentioned in Section 3.4, we used the anomaly scores of the ‘LSTM-1’ model as an example to calculate the anomaly threshold. Figure 4 shows the clustering results of the anomaly scores of the ‘LSTM-1’ model using K-means clustering. As can be seen, the circles in the figure are approximately divided into two groups, with red circles representing anomalous clusters and black circles representing normal clusters, as can be seen. We can see that the normal cluster center was 0.032, the anomaly cluster center was 0.405, and the anomaly threshold was 0.150. Table 4 shows the number of false alarms generated by all the models in the experiment after the anomaly threshold was determined. It can be seen that the number of false alarms decreases with the increase in the moving average window size. This is because the processed data are smoother when the moving average window size is bigger. It is easier to extract data characteristics in the seq2seq model, resulting in superior reconstruction results. Furthermore, we can observe that applying the attention mechanism improves sequence reconstruction. Finally, due to the use of multi-layer convolution stacking, it is possible to capture long-time information. For example, if the convolution kernel size is 4, the first layer of convolution that can cover the maximum length of the original sequence is 4, the second layer of convolution that can cover the maximum length of the original sequence is 16, and so on. These results show that the maximum length that can be covered by the original sequence grows exponentially as the number of convolution layers grows, allowing for the extraction of information over a longer time span. In Table 4, we can see that the CNN-based seq2seq model outperformed the LSTM-based seq2seq model.

The model’s reconstruction ability can be evaluated by the detection ability of point anomaly. In this experiment, 1000-point anomalies with normal distribution were added to the test data to assess the model’s detection capacity. We needed to use the point anomalies as positive samples because our goal was to discover anomalies. Precision and recall rates were determined using a procedure that differed from the standard [1]. Their equations are briefly defined here as:

Precision is given by Equation (6):

(6) $$\mathrm{Precision}\;=\frac{True\;Negatives}{True\;Negatives+False\;Negatives}\times 100,$$

Recall is given by Equation (7):

(7) $$\mathrm{Recall}\;=\frac{True\;Negatives}{True\;Negatives+False\;Positives}\times 100,$$

The experimental results are shown in Table 4. The results show that when the moving average window size increases, the number of false alarms falls at first, then increases, while precision and recall increase, then decline. When the size of the moving average window is set to 4, the model generates the least false alarms while having the highest precision and recall. The reason behind this is that when the data are not smoothed, the model has a harder time extracting the data features, resulting in more false alarms. When data are over-smoothed, the intrinsic features of the data are lost, preventing the model from extracting the features of the real data, resulting in the formation of more false alarms. In addition, we can see that the CNN-based seq2seq models outperform the LSTM-based seq2seq models, and the performance will be better if the attention mechanism is used.

In order to compare the performance between the ST-DAE and seq2seq models proposed in this paper, the minimum size of the hidden layers in the ST-DAE model is set to 2. It can also be seen that the best results produced by the models proposed in this paper have already better than those of the ST-DAE model. This shows that the seq2seq models can achieve the batter reconstruction capability than the denoising autoencoder.

#### 4.4.2. Evaluation on Time-Dependent Anomalies Detection Capability

**Figure 5:**
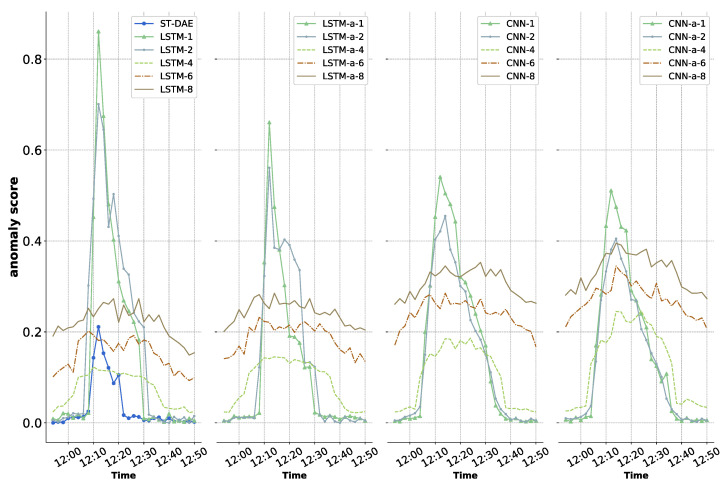
Anomaly scores of the time-dependent anomaly.

When an anomaly arises in the satellite power subsystem, it may take longer to reach full power charging, and this anomaly is a time-dependent anomaly according to Section 3.4. Figure 5 shows the detection results for such an anomaly using the ST-DAE model and 20 seq2seq models. When the moving average window size was 1 or 2, the seq2seq models acquired higher anomaly scores in the early stages of the anomaly, indicating a larger probability of anomalous occurrence. This could be because the data were still fluctuating when the moving average window was set at 1 or 2, and the model was unable to acquire adequate high-level information. Furthermore, this stage occurs at the crossroads of working situation changes, thus, such a high abnormal score is the result of false alarms. In the same case, the CNN-based model produced lower anomaly scores for false alarms than those produced by the LSTM-based model, which indicates that the CNN-based model had a better ability to limit false alarms. The shape of the anomaly score curve changed significantly when the size of the moving average window is 4, 6, or 8. It did not produce excessive anomaly scores in the early stages of the anomaly. This means that when the data were substantially smoothed, the transition of working conditions slowed down, and as the model can manage these smoothed transition period data, the false alarm disappears. However, the anomaly scores have improved overall, and the gap between normal and anomalous samples has narrowed. The figure shows that when the moving average window size is 4, the anomaly scores are overall lower than when the window size is 6 or 8, but the normal and anomalous samples’ anomaly scores are more distinct. This means that we can more easily identify anomalies in the results generated by this model. Among the models in the experiment, CNN-based seq2seq attention models have better performance.

As can be seen in Figure 5, the result produced by the ST-DAE model is the same as the seq2seq model when the moving average window is set to 1 and 2 for the detection of time-dependent anomalies, and only a higher anomaly score is obtained in the early stage of the anomaly. It shows that the traditional non-seq2seq model does not have the ability to detect time-dependent anomalies.

#### 4.4.3. Evaluation on High-Level Features Quality

As described in Section 3.4, the performance of the model is assessed by evaluating the quality of high-level features. In Section 4.3, all models in the experiment extracted two high-level features, which were clustered using the K-means algorithm to obtain the classification results for the four working conditions. The count of misclassified samples was obtained by comparing the obtained classification results with the manually labeled working condition results. The number of error clustering samples for different models and the calculated results of the Silhouette Coefficient Score and the Calinski-Harabasz Index are shown in Table 5.

The results show that when the moving average window size grows, the number of error clustering samples decreases at first, then rises, while the Silhouette Coefficient Score and Calinski-Harabasz Index increase, then decline. The results suggest that the same conclusions can be reached as in Section 4.4.1. This means that proper data smoothing is more beneficial to improving the quality of high-level features. Meanwhile, CNN-based seq2seq models have better performance than the LSTM-based seq2seq models. The highest clustering precision is achieved using the high-level features generated by the CNN-based seq2seq model under the condition that the moving average window size is 4. When the data smoothness level is set too high or too low, the quality of the high-level features suffers. The use of attention mechanism does contribute to the generation of more meaningful high-level features. Meanwhile, it can also be seen that the best results produced by the models proposed in this paper are already better than those of the ST-DAE model. This shows that the seq2seq models have better high-level features extraction capability.

## 5. Conclusions

Anomaly detection in satellite power subsystems can provide significant information for aerospace experts looking to improve satellite operating performance. The seq2seq model is more promising for practical applications, because the telemetry data of satellite power subsystem health monitoring are time series data with few abnormal samples. The seq2seq model not only has all of the advantages of the traditional autoencoder model, but it also has better feature extraction capacity and can handle time-dependent information. The effectiveness of the seq2seq model in detecting anomalies in the satellite power subsystem was investigated in this study. The following is a summary of the primary contribution: (1) For unsupervised anomaly detection, a seq2seq-based scheme was proposed. The scheme was created using various neural network cell types and the moving average method’s window size. To the best of the authors’ knowledge, this was the first attempt at detecting anomalies in the satellite power subsystem. (2) Methods are presented for evaluating the performance of the seq2seq model indirectly. When anomaly labels are unknown, these strategies are quite useful.

The research revealed that the suggested seq2seq-based approach is capable of detecting both common point anomalies and time-dependent types of anomalies. For practical applications, the approach can be fully automated and integrated with the Satellite Operations Management System (OMS) on the ground. The final anomaly scores are straightforward to comprehend. They range from zero to one and can be thought of as anomalous possibilities. The performance of different neural network cell types and moving average window sizes has been assessed and compared. The model’s reconstruction capacity is assessed using the common point anomaly detection task. The model’s capacity to extract time-dependent information is assessed using the time-dependent anomaly detection challenge. A supplemental high-level features clustering task was devised to assess the seq2seq model’s dependability indirectly using the classification precision of the four working conditions. It was demonstrated that the CNN-based seq2seq attention model may better maintain the information encoded in temporal data in terms of the seq2seq architecture. In addition, it was shown that proper data smoothing (e.g., the moving average window size is 4) can enable the seq2seq model to learn more reliable and robust features from the satellite power subsystem telemetry data.

## Figures and Tables

**Table 1:** Summary of seq2seq frameworks.

Seq2seq	Network Cell Type	Attention	N
LSTM-1	LSTM	Without attention	1
LSTM-2	LSTM	Without attention	2
LSTM-4	LSTM	Without attention	4
LSTM-6	LSTM	Without attention	6
LSTM-8	LSTM	Without attention	8
LSTM-a-1	LSTM	With attention	1
LSTM-a-2	LSTM	With attention	2
LSTM-a-4	LSTM	With attention	4
LSTM-a-6	LSTM	With attention	6
LSTM-a-8	LSTM	With attention	8
CNN-1	CNN	Without attention	1
CNN-2	CNN	Without attention	2
CNN-4	CNN	Without attention	4
CNN-6	CNN	Without attention	6
CNN-8	CNN	Without attention	8
CNN-a-1	CNN	With attention	1
CNN-a-2	CNN	With attention	2
CNN-a-4	CNN	With attention	4
CNN-a-6	CNN	With attention	6
CNN-a-8	CNN	With attention	8

**Table 2:** Structures of seq2seq models based on LSTM and LSTM with attention.

	Layer	Description	Size
encoder	1	Input	240 × 36
	2	lstm_1	240 × 18
	3	lstm_2	240 × 9
	4	lstm_3	240 × 2
decoder	5	lstm_4	240 × 2
	6	lstm_5	240 × 9
	7	lstm_6	240 × 18
	8	dense_1	240 × 36

**Table 3:** Structures of seq2seq models based on CNN and CNN with attention.

	Layer	Description	Size
encoder	1	Input	240 × 36
	2	conv_1	240 × 36
	3	maxpool_1	2 × 2
	4	conv_2	120 × 18
	5	maxpool_2	2 × 2
	6	conv_3	60 × 2
decoder	7	conv_4	60 × 2
	8	uppool_1	2 × 2
	9	conv_5	120 × 18
	10	uppool_2	2 × 2
	11	conv_6	240 × 36

**Table 4:** The summary of false alarms and results of point anomaly detection.

Seq2seq	False Alarms	Precision (%)	Recall (%)
ST-DAE [1]	43	90.23	91.58
LSTM-1	83	86.79	87.39
LSTM-2	71	89.40	89.02
LSTM-4	52	91.37	91.81
LSTM-6	77	87.84	88.98
LSTM-8	90	84.37	85.64
LSTM-a-1	72	87.92	88.32
LSTM-a-2	61	89.80	90.88
LSTM-a-4	46	92.74	93.42
LSTM-a-6	67	88.41	89.11
LSTM-a-8	78	86.95	88.05
CNN-1	55	89.78	91.48
CNN-2	44	92.80	93.71
CNN-4	26	94.45	95.95
CNN-6	49	92.17	92.17
CNN-8	59	88.69	90.03
CNN-a-1	42	91.44	92.01
CNN-a-2	36	93.08	94.63
CNN-a-4	10	96.59	98.09
CNN-a-6	37	92.87	93.72
CNN-a-8	49	90.69	91.60

**Table 5:** The results of high-level features clustering.

Seq2seq	Error Clustering Samples	Silhouette Coefficient Score	Calinski-Harabasz Index
ST-DAE [1]	77	0.8953	5362
LSTM-1	112	0.7972	5065
LSTM-2	95	0.8704	5732
LSTM-4	85	0.9040	5904
LSTM-6	103	0.8497	5647
LSTM-8	125	0.7201	4893
LSTM-a-1	104	0.8323	5251
LSTM-a-2	97	0.9062	5923
LSTM-a-4	76	0.9211	6201
LSTM-a-6	94	0.8975	5854
LSTM-a-8	115	0.7831	5034
CNN-1	63	0.8834	5748
CNN-2	48	0.9266	6343
CNN-4	30	0.9451	7113
CNN-6	50	0.9055	6042
CNN-8	62	0.8609	5433
CNN-a-1	53	0.9029	6128
CNN-a-2	32	0.9336	6732
CNN-a-4	16	0.9615	7537
CNN-a-6	34	0.9305	6326
CNN-a-8	56	0.8913	5735

## Data Availability

Not applicable.
